# Dynamics of a Perturbed Microbial Community during Thermophilic Anaerobic Digestion of Chemically Defined Soluble Organic Compounds

**DOI:** 10.3390/microorganisms6040105

**Published:** 2018-10-11

**Authors:** Luka Šafarič, Sepehr Shakeri Yekta, Tong Liu, Bo H. Svensson, Anna Schnürer, David Bastviken, Annika Björn

**Affiliations:** 1 Department of Thematic Studies-Environmental Change, Linköping University, 581 83 Linköping, Sweden; luka.safaric@liu.se (L.Š.); bo.svensson@liu.se (B.H.S.); anna.schnurer@liu.se (A.S.); david.bastviken@liu.se (D.B.); annika.bjorn@liu.se (A.B.); 2 Biogas Research Center, Linköping University, 581 83 Linköping, Sweden; 3 Department of Molecular Science, Swedish University of Agricultural Science, Uppsala BioCenter, 75007 Uppsala, Sweden; tong.liu@slu.se

**Keywords:** Thermophilic Anaerobic Digestion, process perturbation, process stability, microbial community dynamics, trace elements

## Abstract

Knowledge of microbial community dynamics in relation to process perturbations is fundamental to understand and deal with the instability of anaerobic digestion (AD) processes. This study aims to investigate the microbial community structure and function of a thermophilic AD process, fed with a chemically defined substrate, and its association with process performance stability. Next generation amplicon sequencing of 16S ribosomal RNA (rRNA) genes revealed that variations in relative abundances of the predominant bacterial species, *Defluviitoga tunisiensis* and *Anaerobaculum hydrogeniformans,* were not linked to the process performance stability, while dynamics of bacterial genera of low abundance, *Coprothermobacter* and *Defluviitoga* (other than *D. tunisiensis*), were associated with microbial community function and process stability. A decrease in the diversity of the archaeal community was observed in conjunction with process recovery and stable performance, implying that the high abundance of specific archaeal group(s) contributed to the stable AD. Dominance of hydrogenotrophic *Methanoculleus* particularly corresponded to an enhanced microbial acetate and propionate turnover capacity, whereas the prevalence of hydrogenotrophic *Methanothermobacter* and acetoclastic *Methanosaeta* was associated with instable AD. Acetate oxidation via syntrophic interactions between *Coprothermobacter* and *Methanoculleus* was potentially the main methane-formation pathway during the stable process. We observed that supplementation of Se and W to the medium improved the propionate turnover by the thermophilic consortium. The outcomes of our study provided insights into the community dynamics and trace element requirements in relation to the process performance stability of thermophilic AD.

## 1. Introduction

Process stability is a basic requirement for industrial application of anaerobic digestion (AD) as a reliable technology for organic waste treatment and biogas production. Stable operation of AD depends on coordinated activities of microorganisms that are responsible for a cascade of organic matter degradation pathways, which include hydrolysis, fermentation, anaerobic oxidation, and methanogenesis [1]. A sufficient supply of energy sources, macro- and micronutrients is needed in order to establish a functionally resilient microbial community structure in AD processes, and to ensure a balanced carbon and electron flow through the anaerobic degradation chain [2,3]. However, process perturbations may emerge, e.g., due to varying substrate composition and loading rate, occurrences of inhibitory compounds, or the deficiency of essential nutrients, impeding the stable operation of AD [4]. Process perturbations often disrupt the kinetic synergies among the metabolic reactions, promoting alterations in microbial community structure and function [4,5]. It is generally recognized that process instabilities are more frequent during the operation of thermophilic AD (operational temperature of ca 55 °C) compared to mesophilic AD (operational temperature of ca 37 °C). This is commonly attributed to a comparatively lower diversity of active members in thermophilic microbial cultures in AD reactors, implying a relatively lower degree of functional redundancy, which makes the metabolic association of different microorganisms more sensitive to disturbances [6,7,8]. Concerns regarding the process stability of thermophilic AD demotivate its widespread application, despite potential advantages, such as high organic matter conversion kinetics, low substrate retention times, and possibilities for pathogen removal [9,10]. In this context, an understanding of the microbial community dynamics in relation to process perturbations is fundamental for dealing with instabilities of AD processes, particularly for those operated under thermophilic conditions.

In a previous study (cf. Speda et al. [11]), a chemically defined substrate was used to enrich a microbial culture that was capable of metabolizing intermediate degradation products, which commonly occur during AD processes (i.e., fermentation of soluble carbohydrate and protein subunits, and anaerobic oxidation of fatty acids and alcohols, as well as methanogenesis). Furthermore, operational conditions for thermophilic AD of the chemically defined substrate in a laboratory continuous stirred tank reactor (CSTR) were optimized (i.e., substrate loading rate and retention time) to ensure a constant conversion rate of the substrate to biogas. As a result, a stable thermophilic AD process was operated for several years with constant biogas production and substrate-conversion rates, implying the establishment of a thermophilic microbial culture at a metabolic steady state [12]. In an attempt to reproduce the thermophilic AD process in a new reactor by using the aforementioned microbial culture, we faced difficulties to stabilize the process. Although operational conditions during the startup phase (i.e., the process temperature, substrate composition, substrate loading rate, and retention time) were similar to the original reactor, instability of the AD process occurred shortly after transferring the culture from the original CSTR with a 9 L working volume to a new CSTR with a 4 L working volume.

A series of remedy measures based on theoretical knowledge and earlier experiences were implemented, which led to a recovery of the anaerobic digester’s functionality, followed by reoccurring process instabilities and failure after 500 days of operation. These operational phases provided a suitable case study to assess the dynamics of the microbial community in relation to process performance stability and perturbations. Accordingly, the aim of our study was to investigate the microbial community structure, and the function of the thermophilic AD process during disturbances and process stability. The objectives to achieve this aim were the evaluation of process performance by measuring biogas and methane production, pH, and volatile fatty acid (VFA) concentrations, together with an assessment of microbial community dynamics using high-throughput sequencing of bacterial and archaeal 16S ribosomal RNA (rRNA). We believe that the outcomes of our study contribute to a better understanding of the behavior and functional capability of thermophilic microbial communities upon emergence of process perturbations during thermophilic AD, with potential implications for process control and optimization.

## 2. Materials and Methods

A laboratory-scale CSTR was inoculated by transferring 4 L of microbial culture from a thermophilic anaerobic digester, which had operated at steady state conditions for three years. Operational conditions and substrate composition of the new thermophilic reactor were identical to the original reactor, except for the working volume, which was decreased from 9 to 4 L. The chemically defined substrate consisted of glucose, sucrose, acid-hydrolyzed casein, ethanol, methanol, acetic acid, propionic acid, butyric acid, and formic acid as carbon and energy sources, together with essential macro- and micronutrients. Detailed information on the composition of the substrate and the concentrations of individual components is presented in Table S1. The initial organic loading rate (OLR) was 1.9 g L^−1^ day^−1^ chemical oxygen demand (COD) at a hydraulic retention time (HRT) of 30 days, i.e., 133 mL of reactor material, was exchanged with the chemically defined substrate every day. The OLR was further adjusted to alleviate process disturbances over the 500 days of reactor operation (see Section 3.1). The reactor was initially operated at 55 ± 1 °C until the temperature was further adjusted to 52 ± 1 °C at day 60 to attenuate potential inhibitory effects of free ammonia in the medium [13,14]. Biogas production was monitored by a gas meter working by the principle of liquid displacement (Ritter GmbH, Bochum, Germany), and it is presented for normal conditions (0 °C and 1.013 bar). The methane concentration of the biogas was measured online with a sensor based on infrared light absorption (BlueSens gas sensor GmbH, Herten, Germany), and the daily average values were reported. The pH was measured by a pH meter (InoLab 7310, WTW, Weilheim, Germany), and concentrations of VFA were determined by gas chromatography according to Jonsson & Boren [15]. Total solid (TS) and volatile solid (VS) contents of the reactor medium were determined according to the Swedish Standard method (SS-028113).

Biomass samples were regularly collected for bacterial and archaeal community composition analyses and stored at 20 °C. Samples, collected at days 2, 10, 101, 129, 178, 214, 228, 260, 304, 332, 381, and 472, were used for DNA extraction. DNA was isolated with the UltraClean Microbial DNA isolation kit (Mo Bio Laboratories Inc., Carlsbad, CA, USA) according to the manufacturer’s instruction, and stored in nuclease-free water at −20 °C. Glass beads (Glass Bead Tubes 0.1 mm, Mo Bio Laboratories Inc., Carlsbad, CA, USA) were used instead of the Garnet beads from the kit, for improving cell lysis and DNA extraction yield [16]. The DNA extracts were processed for high-throughput amplicon sequencing of 16S rRNA genes by the Illumina MiSeq platform (Illumina Inc., San Diego, CA, USA) at the SNP&SEQ Technology platform in Uppsala, Sweden. The universal primer set 515’F and 805R was used for general amplification of 16S rRNA genes [17], and the specific primer set 516F and 915R was used for amplification of archaeal 16S rRNA genes [18]. Readers are referred to Liu et al. [19] and Westerholm et al. [20] for details on the amplification procedure prior to high-throughput sequencing (i.e., two-step PCR settings). The raw sequencing data was processed by the open-source software DADA2, in which amplicon sequence variants (ASV) are used for taxonomic assignments [21,22]. Details on data processing steps and the number of bacterial and archaeal sequences acquired from the DADA2 pipeline are presented in the supplementary information in text S1 and Tables S2 and S3. The taxonomy assignments were acquired using the database SILVA (release 132) [23]. The raw sequencing data is available at National Center for Biotechnology Information (NCBI) database with identification number: SRP149871. Bacterial and archaeal diversities were assessed by calculating Hill diversity numbers at two orders; ^0^*D* and ^1^*D*; based on ASV read counts, which were suggested as the most relevant indices for diversity assessments [24,25]. The similarity/dissimilarity of the bacterial and archaeal sequence reads among samples collected at different days was evaluated by hierarchical cluster analysis of the Bray–Curtis distance of the sequencing results. The sequencing data was standardized using the Hellinger method [26] prior to calculation of the Bray–Curtis distances, and the computation was performed in R [27], by functions available in the vegan package [28].

## 3. Results and Discussion

### 3.1. Process Startup, Recovery, and Failure

**Figure 1:**
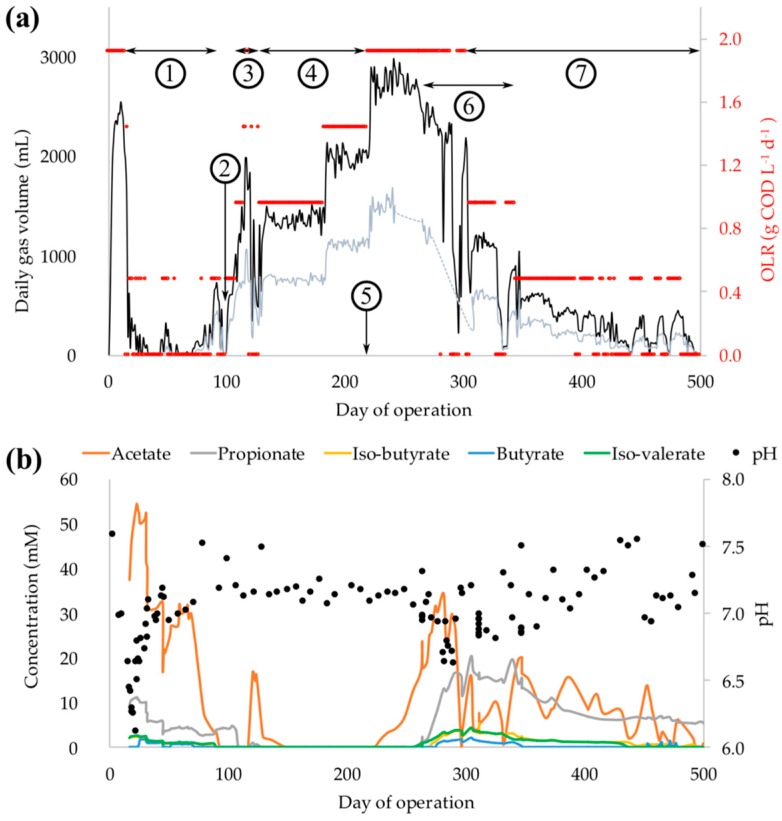
(**a**) Daily production of biogas (black line) and methane (grey line; dotted when data is missing) together with organic loading rates (OLR) of the reactor. Digits mark the times when operational conditions of the reactor were altered 1: Addition of buffer medium, dilution of volatile fatty acids, and OLR adjustment. 2: Increase in influent Se and W concentration. 3: Adjustment of OLR. 4: Stepwise increase of OLR to target value of 1.9 g COD L^−1^ day^−1^. 5: Accidental overdose of Se and W. 6: Increase in influent Ni concentration. 7: Stepwise decrease of OLR and reactor failure. (**b**) Volatile fatty acids concentrations and pH of the reactor medium.

Upon process start-up, biogas production reached 2500 ± 75 mL (corresponding to 330 ± 10 mL g^−1^ COD^−1^), at day 11, followed by a rapid decline to zero (Figure 1a). Acetate and propionate accumulated, lowering the pH from 7.6 to 6.1 (Figure 1b), which is below the typical pH range of 6.5 to 7.5 for optimum growth of methanogens in anaerobic digesters [29]. To alleviate the acidification of the reactor medium, the daily OLR was lowered from 1.9 to 0.48 g COD L^−1^ day^−1^, while the HRT was kept constant by diluting the substrate with a buffer solution (20 mM KH_2_PO_4_, 21 mM Na_2_HPO_4_, and 61 mM NaHCO_3_). Furthermore, substrate feeding was occasionally discontinued to prevent further VFA accumulation, while only the buffer solution was supplemented to the reactor. Since the biogas production did not resume, the stirring was turned off to allow for a partial sedimentation of the suspended cells, and 1.8 L of reactor content from the top layer was exchanged by 2 L of buffer solution. As a result, the pH increased from 6.1 to 7.1, and acetate and propionate concentrations decreased from 54 to 31 mM and 11 to 6 mM, respectively (Figure 1b). After the pH adjustment, daily feeding of the reactor was stopped at four occasions (days 33 to 37, 39 to 43, 53 to 56, and 60 to 64) to allow the microorganisms to consume the remaining acetate and propionate. However, the acetate and propionate concentrations did not change, and methane concentration of the biogas remained < 30%, indicating that fermentation took place, while anaerobic oxidation of the acids and methanogenesis were still hampered. High VFA concentrations in the medium may inhibit the AD processes due to permeation of the undissociated form of acids across the cell membrane, and the consequent change of the intracellular pH [30]. Thus, we decided to dilute the VFA contents of the reactor. On several occasions between days 65 and 75, we removed and centrifuged 200 mL of the reactor medium, suspended the cells in 200 mL buffer solution, and returned the suspension into the reactor, which allowed the acetate and propionate concentrations to decrease to below 17 and 3 mM, respectively (Figure 1b). Anaerobic conditions were ensured throughout this procedure by continuously flushing the tubes and bottles with nitrogen gas and using deaerated ultrapure water for preparation of the buffer solution. Thereafter, feeding was resumed at an OLR of 0.48 g COD L^−1^ day^−1^, the acetate concentration started to decline, and biogas production increased from zero to approximately 730 mL (Figure 1a), with methane concentrations of 61 %, on day 92 (daily methane production of 445 mL). The theoretical methane potential from 0.48 g COD L^−1^ day^−1^ of the substrate is 670 mL, which indicates that the process reached a methane production yield of 66% of the theoretical value.

Despite improvement of the biogas production, propionate concentration increased from 3 to 5 mM between days 80 and 100 (Figure 1b). Propionate accumulation in AD processes may be related to deficiency of trace elements, which are essential for syntrophic propionate oxidation and hydrogenotrophic methanogenesis [31,32]. Enzymes containing Se, W, and/or Mo are particularly involved in metabolic reactions during propionate oxidation (e.g., formate dehydrogenases and hydrogenases) [33]. Furthermore, it has been demonstrated that the metabolic functions of Mo and W are analogous due to their similar chemistry, whereas W commonly occurs in thermophilic anaerobic microorganisms [34]. Therefore, we decided to increase the concentration of Se and W in the substrate from 0.08 to 0.16 nM at day 101 and onward, to promote syntrophic propionate oxidization. As a result, propionate concentration rapidly dropped below the detection limit of the analysis (0.2 mM; Figure 1b), which signifies that Se and/or W deficiency contributed to the instability of the process. Previous studies have reported that supplementation of trace metal cocktails, containing different combinations of Fe, Co, Ni, Mo, Se, and W, may lead to an improvement of the overall process stability of thermophilic AD [35]. Our observations particularly point out the significance of Se and/or W availability (among other metals) for attenuating process instabilities that are associated with a suppressed propionate oxidation pathway during thermophilic AD processes.

Subsequent to the decline in acetate and propionate concentrations, we increased the OLR to 1.9 g COD L^−1^ day^−1^, which resulted in an accumulation of acetate (day 120 in Figure 1b) and a rapid drop of the daily methane production from 1065 to 749 mL (Figure 1a). Furthermore, methane production yield declined from 53% to 28% of the theoretical value, which together with the increasing acetate concentration, indicated a higher rate of acetate formation compared to the rate of its consumption in relation to the OLR of the process, e.g., due to an improved propionate oxidation rate after increasing Se and W concentrations. Thus, the OLR was lowered to 0.96 g COD L^−1^ day^−1^ and consequently, the acetate concentration declined to zero. Between days 129 and 219 (i.e., 3 HRT), the reactor performance was stable at OLR of 0.96 and 1.43 g COD L^−1^ day^−1^ with no signs of process disturbances. This implies that sufficient supply of nutrients and proper operational conditions (e.g., OLR and HRT) were ascertained, i.e., balanced kinetics amongst metabolic reactions during AD occurred. Furthermore, the initial process failure as well as pH adjustment and dilution of VFA by the buffer solution resulted in a partial removal of the biomass, which was evident from the decrease in total volatile solids (TVS; % of total mass) of the reactor medium from 0.4% to 0.2% between days 10 and 100. Along with the process recovery, the TVS increased up to 0.6%, indicating a reestablishment of microbial biomass growth.

After a period of stable operation, the OLR of the reactor was reset to the initial value of 1.9 g COD L^−1^ day^−1^ and sustained at this level between days 220 and 302, i.e., for ca 3 HRT (Figure 1a). This resulted in a gradual accumulation of acetate, followed by an increase in propionate concentration (Figure 1b). It was therefore obvious that the consumption rate of acetate was the primary rate-limiting factor of AD at an elevated OLR. As a remedy measure, we increased the concentration of Ni from 0.13 to 0.40 nM in the substrate. Nickel-containing enzymes are involved in various metabolic reactions, such as acetoclastic, hydrogenotrophic, and methylotrophic methanogenesis (e.g., in methyl coenzyme M reductase, F_420_-reducing hydrogenase, and acetyl-CoA synthase) [36]. Accordingly, Ni deficiency may impede an efficient acetate consumption and hamper syntrophic interactions, which are dependent on hydrogen consumption by hydrogenotrophic methanogens. However, extra Ni supplementation had no apparent effect on the process performance, implying that Ni deficiency was not the reason behind the process perturbations at this stage. Extra Ni supplementation was discontinued from day 337. The OLR was decreased again from 1.9 to 0.48 g COD L^−1^ day^−1^. However, the process performance continued to deteriorate and it could not be recovered. The reactor was accidentally supplemented with extra Se and W at day 220, which corresponded to 30-times higher concentrations of these metals inside the reactor. Nevertheless, no immediate response in the form of changes in biogas production or VFA concentrations could be linked to process stimulation or the acute toxicity of microorganisms due to the overdose of Se and W.

### 3.2. Dynamics of the Bacterial Community Composition

**Figure 2:**
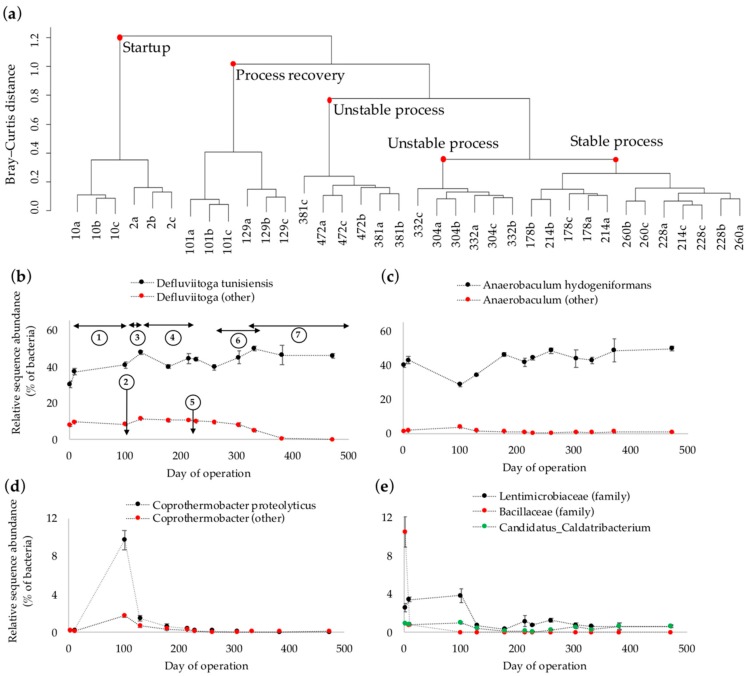
(**a**) Hierarchical cluster analysis of similarity/dissimilarity (Bray-Curtis distance measure) of the prokaryotic sequence reads as determined by next generation amplicon sequencing of 16S rRNA genes from the samples collected at days 2, 10, 101, 129, 178, 214, 228, 260, 304, 332, 381, and 472. Replicate high-throughput sequencing analyses are marked by a, b, and c. (**b–e**) Changes in relative sequence abundances of dominant bacteria (relative abundance >1% of bacteria). Digits in (**b**) mark the times when operational conditions of the reactor were altered (see Figure 1 caption).

The zero order Hill diversity (^0^*D*) of bacterial ASV reads, representing the community richness [24], had a decreasing trend from day 0 to 300, followed by a moderate increase along with process deterioration and failure towards the end of operation (Figure S1). The first order Hill diversity number (^1^*D*) rapidly declined after the startup of the process from day 2 to 10 (Figure S1). The remedy measures applied for pH adjustment and VFA dilution between days 10 and 101 resulted in an increase in ^1^*D*, which however, declined again to a relatively constant level at day 228 and onward (Figure S1). Accordingly, it is likely that an increase in OLR and the observed process performance recovery after day 100 (cf. Figure 1a) resulted in a selective enrichment of specific microbial groups and consequently decreased the diversity of the microbial community. Hierarchical cluster analysis of the prokaryotic sequence reads further revealed dissimilar community compositions between the groups of samples collected: i. at startup of the reactor (days 2 and 10), ii. during process recovery (days 101 and 129), iii. during stable operation (days 178, 214, 228 and 260), and iv. during process instability towards the end of the reactor operation (days 304, 332, 381, and 472; Figure 2a). A substantially different community composition at the startup phase (day 2) signifies an alteration of the microbial community during the experiment (Figure 2a). Based on taxonomic assignments, prokaryotic sequence reads of the initial samples collected at day 2 encompassed 96% bacteria, largely represented by the phyla Synergistetes (42%), Thermotogae (38%), Firmicutes (12%), Atribacteria (3%), and Bacteroidetes (1%), as well as 4% archaea of the Euryarchaeota phylum. Synergistaceae and Petrotogaceae families, represented mainly by *Anaerobaculum hydrogeniformans* (40% of bacteria) and *Defluviitoga tunisiensis* (30% of bacteria), had the highest relative abundances among the bacteria.

*D. tunisiensis* uses a wide range of carbohydrates as electron donors, but not peptone, ethanol, or acetate [37]. The main products of the glucose metabolism by *D. tunisiensis* are acetate, H_2_, and CO_2_. This species commonly occurs in thermophilic AD processes [38], likely in metabolic association with hydrogenotrophic methanogens [39]. The sucrose and glucose in the chemically defined substrate might therefore promote the growth and enrichment of *D. tunisiensis* in the thermophilic microbial culture. The relative abundance of this species varied between 30% and 53% of bacteria, but the disturbances that occurred during the operation did not seem to have an apparent effect on the relative abundance of *D. tunisiensis* (Figure 2b). On the other hand, undefined species of the *Defluviitoga* genus decreased in relative abundance from 10% to 0% of bacteria, in parallel with process failure, making it the only bacterial group that showed a decline in relative abundance towards the end of reactor operation (Figure 2b). Therefore, it may be speculated that the undefined species of *Defluviitoga* genus played an important role in substrate metabolism and process stability, since a decline in their relative abundance was associated with process failure.

*A. hydrogeniformans* contributed to 29–50% of bacteria (Figure 2c). *A. hydrogeniformans* ferments amino acids and a limited number of carbohydrates to acetate, H_2_, and CO_2_, and has an optimum growth temperature of 55 °C and pH of 7.0 [40]. The relative abundance of *A. hydrogeniformans* temporarily decreased between days 10 and 101, during which the addition of buffer, dilution of VFA, and OLR adjustment were applied for process recovery (Figure 2c). Subsequently, an improved process performance in terms of low VFA concentration, stable pH, and recovery of biogas production, led to an increase in relative abundance of *A. hydrogeniformans*, which remained relatively constant towards the end of reactor operation (Figure 2c). Other bacteria with relative abundances >1%, in at least one sampling occasion, were undefined species of *Coprothermobacter* and *Anaerobaculum* genera, *Coprothermobacter proteolyticus*, and the *Candidatus Caldatribacterium* genus as well as families of Lentimicrobiaceae and Bacillaceae (Figure 2d,e). The relative abundance of *Coprothermobacter* genus, together with unspecified species of *Anaerobaculum* temporarily increased from 0.0% to 12%, and 2.0% to 4.0% of bacteria at day 101, respectively. The growth substrates of *Coprothermobacter* genus are mainly proteins (e.g., peptone and casein), while carbohydrates (e.g., glucose and sucrose) are weakly utilized in the absence of medium additives, such as yeast extract [41,42]. Accordingly, an increase in relative abundance of *Coprothermobacter* and unspecified species of *Anaerobaculum* might be linked to the simultaneous decrease in abundance of the most dominant protein-fermenting species, *A. hydrogeniformans*, and an enhanced protein availability in the medium. Bacillaceae, whose function in AD processes is associated with hydrolysis and fermentation of carbohydrates [43,44], was the only bacterial group that showed a substantial decrease in relative abundance directly after startup of the reactor (from 11% to 1.0% of bacteria; Figure 2e).

### 3.3. Dynamics of the Archaeal Community Composition

**Figure 3:**
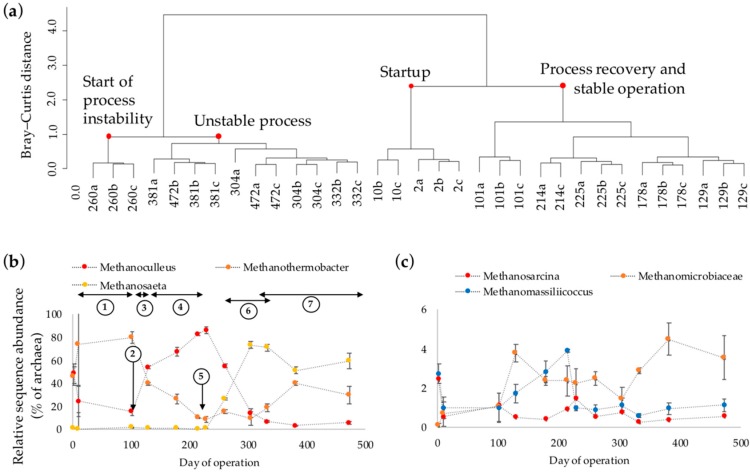
(**a**) Hierarchical cluster analysis of similarity/dissimilarity (Bray–Curtis distance measure) of the archaeal sequence reads as determined by next generation amplicon sequencing of 16S rRNA genes from the samples collected at days 2, 10, 101, 129, 178, 214, 228, 260, 304, 332, 381, and 472. Replicate high-throughput sequencing analyses are marked by a, b, and c. (**b**,**c**) Changes in the relative sequence abundances of dominant archaea (relative abundance >1% of archaea). Digits in (**b**) mark the times when operational conditions of the reactor were altered (see Figure 1 caption).

The relative abundance of archaeal phylum Euryarchaeota rapidly declined after the startup of the process from 4.0 (day 2) to 2.7 (day 10) and further to <1.0% of prokaryotic sequences towards the end of experiment. The archaeal richness, as represented by zero order Hill diversity (^0^*D*) for archaeal 16S rRNA profiles (Figure S2), showed an increasing trend over the course of the experiment. Interestingly, archaeal diversity (^1^*D*) declined upon process performance recovery, while it increased along with the emergence of process instabilities towards the end of the operation (Figure S2). Thus, specific archaeal group(s) likely prevailed during process recovery and stable performance, since diversity of the archaeal community declined under stable operation of the reactor. Furthermore, the hierarchical cluster analysis of the archaeal sequence reads showed that the archaeal community composition was different during process perturbations, as compared to periods of process recovery and stability (Figure 3a). As for the bacteria, the archaeal composition in the initial sample differed from those collected over the course of the experiment (Figure 3a). Initially, Methanomicrobiaceae and Methanobacteriaceae families, represented by the hydrogenotrophic *Methanoculleus* and *Methanothermobacter* genera (48% and 46% of archaea, respectively), dominated the culture. Both genera mainly convert carbon dioxide and hydrogen to methane [45,46,47]. Acetoclastic *Methanosarcina* and *Methanosaeta*, as well as methylotrophic *Methanomassiliicoccus*, were present at relatively low abundances in the initial culture (2.5%, <1.0%, and 2.7% of archaea, respectively). After process startup, the relative abundance of *Methanoculleus* decreased from 48% to 24% of archaea and *Methanothermobacter* dominated the archaeal community (74% of archaea at day 10; Figure 3b). Subsequent to the addition of Se and W (day 101) and a stepwise increase in OLR (between days 130 and 220), the biogas production increased in conjunction with a gradual dominance of *Methanoculleus* over *Methanothermobacter*. The relative abundance of *Methanothermobacter* decreased from 80% to 9% of archaea and *Methanoculleus* dominated (up to 87%) the archaeal community during the stable process performance of the reactor (Figure 3b).

The poor capacity of the microbial consortia to convert acetate, which caused a rapid accumulation of acetate 10 days after startup of the reactor (cf. Figure 1b), might be related to the initially low abundance of the acetoclastic methanogens, *Methanosarcina* and *Methanosaeta* (Figure 3b). However, the relative abundance of the acetoclastic methanogens were low (<2.0% of archaea) even during the stable process performance between days 130 and 220, when the archaeal community was mainly dominated by the hydrogenotrophic *Methanoculleus* (Figure 3b)*.* Although activities of the low-abundant acetoclastic methanogens under stable performance of the process are unknown, predominance of hydrogenotrophic *Methanoculleus*, together with an absence of acetate (<detection limit of 0.2 mM) in the medium suggest the occurrence of the syntrophic acetate oxidation (SAO) pathway. The *Methanoculleus* genus has been reported as the most frequent syntrophic partner of the acetate-oxidizing bacteria typically in mesophilic AD processes [39,48,49]. SAO commonly prevails during inhibition or during low activities of acetoclastic methanogens e.g., due to ammonium inhibition, whereas high temperatures during AD favor methane formation via the association of SAO and hydrogenotrophic methanogenesis, and it can help stabilize perturbed AD systems [13,50]. Among the bacterial groups identified in the reactor, the *Coprothermobacter* genus could potentially contribute to acetate conversion via SAO during the stable process performance. This genus belongs to Thermodesulfobiaceae family, which includes the thermophilic syntrophic acetate oxidizing species of *Thermoacetogenium phaeum* [51], as well as other acetogenic strains such as *Moorella thermoacetica* [52] and *Carboxydothermus hydrogenoformans* [53]. The involvement of *Coprothermobacter* in SAO during thermophilic AD processes was previously suggested [54,55], and acetate oxidation via syntrophic association of *Coprothermobacter* with *Methanothermobacter* was reported for a high temperature (65 °C) anaerobic digester [56]. Moreover, acetate started to accumulate in the reactor from day 230 after increasing the OLR to 1.9 g COD L^−1^ day^−1^ (cf. Figure 1a), when the abundance of *Coprothermobacter* declined from 12% to 0.0% of bacteria (days 101 to 228). This was followed by a decline in *Methanoculleus* abundance from 87% to 6.0% of archaea (days 228 to 472). Thus, the coupled dynamics of *Methanoculleus* and *Coprothermobacter* suggest their potential metabolic association via the SAO pathway.

Accumulation of acetate from day 230 and onward apparently promoted the growth of acetoclastic *Methanosaeta*, with an increase in relative abundance from 0.0% to 73% of archaea. The increase in relative abundance of *Methanosaeta* was accompanied by a decline in acetate and propionate concentrations (Figure 1a). *Methanosaeta* became the dominant genus, which, together with *Methanothermobacter*, were the most abundant archaeal genera during the period leading to process failure at the end of reactor operation. It is noteworthy that we observed a predominance of *Methanosaeta* over *Methanosarcina* (>50% as opposed to <1% of archaea, respectively) at acetate concentrations up to 20 mM (Figure 3b,c). It is well known that the acetoclastic methanogenesis by *Methanosaeta* prevails at low concentration of acetate (<1 mM), while *Methanosarcina* is commonly reported to dominate at high acetate levels [57,58,59]. However, similar to our observations, previous studies reported the unusual competitiveness of *Methanosaeta* over *Methanosarcina* at high acetate concentrations [60]. A specific reason behind the inconsistent behavior of these genera was not reported, but it was argued that differences in nutrient requirements by *Methanosaeta* and *Methanosarcina* under certain conditions might contribute to the predominance of the former at high acetate levels. Furthermore, it has been demonstrated that high hydrogen partial pressure inhibits methane-formation from acetate by certain strains of thermophilic *Methanosarcina*, but not by *Methanothrix* sp. (renamed *Methanosaeta* sp.) [61]. In the present study, it is therefore likely that a higher partial pressure of hydrogen was established in the reactor, as manifested by an accumulation of propionate from day 270 and onward (Figure 1b), which may have in turn provided a competitive advantage for *Methanosaeta* over *Methanosarcina* for acetate uptake. 

## 4. Conclusions

The outcomes of the present study showed that the steady metabolic state of the thermophilic microbial consortia was upset by the transfer to a new reactor with a lower working volume, which impeded an establishment of a stable AD process. Different groups of methanogenic archaea were dominant at different stages of the reactor operation. Taxonomic assignment of archaeal sequences revealed that dominance of *Methanoculleus* in the archaeal community could particularly be associated with process stability, and efficient acetate and propionate oxidation by the thermophilic consortium. However, other methanogens, particularly *Methanothermobacter* and *Methanosaeta*, dominated the archaeal community under unstable process performance. Although *D. tunisiensis* and *A. hydrogeniformans* had the highest relative abundances among the bacterial groups, dynamics of less abundant species (e.g., *Coprothermobacter proteolyticus* and undefined species of *Defluviitoga*) could be related to the process perturbation events. This observation highlights the possible association of low-abundance bacteria, as identified by high-throughput sequencing of bacterial 16S rRNA genes, with process stability, as well as their importance for sustaining the overall function of the microbial community during AD processes. Furthermore, the results suggested that species of *Coprothermobacter* genera might be involved in acetate oxidation via syntrophic interaction with *Methanoculleus*, as the major pathway of methane-formation during the stable performance of the thermophilic AD process. The availability of Se and/or W was apparently critical for achieving a stable process performance, denoting that a sufficient supply of these metals needs to be ensured (e.g., via supplementation) for optimization of the thermophilic AD processes.

## Supplementary Materials

The following supplementary elements are available online at http://www.mdpi.com/2076-2607/6/4/105/s1, Text S1: Next generation amplicon sequence data processing, Table S1: composition of the chemically defined substrate, Table S2: number of bacterial sequence reads remaining for each sample at each step of the DADA2 pipeline, Table S3: number of archaeal sequence reads remaining for each sample at each step of the DADA2 pipeline, Figure S1: Hill diversity indices ^0^*D*, and ^1^*D* for bacterial 16S rRNA profiles, Figure S2: Hill diversity indices ^0^*D*, and ^1^*D* for archaeal 16S rRNA profiles, Supplementary references.
