# Supplementary material for: Dynamics of a Perturbed Microbial Community during Thermophilic Anaerobic Digestion of Chemically Defined Soluble Organic Compounds
Source: Microorganisms. 2018 Oct 11;6(4):105. doi: 10.3390/microorganisms6040105 (PMC6313639; doi:10.3390/microorganisms6040105)
Supplement: Supplementary file 1 [file microorganisms-06-00105-s001.pdf]

microorganisms

Supplementary elements:

Text S1: Next generation amplicon sequence data processing

Table S1. Composition of chemically defined substrate.

Table S2. Number of prokaryotic sequence reads remaining for each sample at each step of the DADA2 pipeline.

Table S3. Number of archaeal sequence reads remaining for each sample at each step of the DADA2 pipeline.

Figure S1. Hill diversity indices ^{0}D, and ^{1}D for bacterial 16S rRNA profiles.

Figure S2. Hill diversity indices ^{0}D, and ^{1}D for archaeal 16S rRNA profiles.

Supplementary references.

Text S1: Next generation amplicon sequence data processing

The adapters were trimmed from the raw sequences with Cutadapt (Martin, 2011). Sequences lacking adapters were removed, as were sequences that did not have a length between 200 and 300 bp or between 250 and 500 bp for primer pairs 515′F/805R and 516F/915R, respectively. Those containing unspecified (i.e. N) bases were removed as well. The number of sequences after this step are presented as *Input* values in Tables S2 and S3.

Further data processing was performed in R with the DADA2 package (Callahan, 2017), where sequence read quality profiles were first inspected, and the sequences filtered with the filterAndTrim function. Based on their quality scores, 515′F/805R sequences were truncated to 220 and 170 bp for forward and reverse sequence reads, respectively, while sequences amplified by 516F/915R were truncated to 245 and 155 bp for forward and reverse sequence reads. The first 35 bases from reverse reads in amplicons from primers 515′F/805R were also trimmed due to low quality scores. In both cases, the maximum expected errors, were set to 2, and all sequences were truncated when their quality score dropped to 11 or lower. The number of remaining sequences is presented as *Filtered* in Tables S2 and S3.

The model of error rates was learned from the data, followed by dereplication of the sequences. Sample inference was performed, resulting in the number of sequences, listed as *Denoised* in Tables S2 and S3, followed by merging of the paired ends. A sequence table was then constructed and the chimeras removed by selecting the "consensus" method. This resulted with the number of sequences presented as *Nochim.* in Tables S2 and S3. At this point, the data was ready to be further analysed for taxonomy assignments with the Phyloseq package (McMurdie and Holmes, 2013).

Table S1. Chemically defined substrate composition. Compounds written in blue correspond to the buffer medium, green to carbon sources, yellow to vitamins, and red to trace elements. The stock solution was prepared by dissolving all the compounds in ultrapure water. It was then diluted to the final concentration by tap water in order to achieve the desired hydraulic retention time. The concentration of elements in the substrate originating from tap water are written in purple.

Substance	Chemical formula	Concentration in stock solution (mM)	Concentration in substrate (mM)
Monopotassium Phosphate	KH_2PO_4/H_2KO_4P	20	13
Sodium Bicarbonate	$NaHCO_3$	61	39
[a]Sodium Sulphate	Na_2SO_4	0.7	0.5
Ammonium Chloride	NH_4Cl	9.4	6.0
Sodium Chloride	NaCl	17	11
Magnesium Chloride Hexahydrate	$MgCl_2$ x $6H_2O$	2.0	1.3
Iron Chloride Tetrahydrate	$FeCl_2$ x $4H_2O$	0.3	0.2
Disodium Phosphate	Na_2HPO_4	21	14
Glucose	$C_6H_{12}O_6$	194	124
Sucrose	$C_{12}H_{22}O_{11}$	41	26
[a]Casein	$C_{38}H_{57}N_9O_9$	25	16
Methanol	CH_3OH	31	20
Ethanol	C_2H_6O	20	13
Acetic Acid	CH_3COOH	17	11
Propionic Acid	$C_3H_6O_2$	2.7	1.7
Butyric Acid	$C_4H_8O_2$	1.1	0.7
Formic Acid	CH_2O_2	2.2	1.4
Biotin (Vit. B8)	$C_{10}H_{16}N_2O_3S$	8.2E-05	1.1E-07
Vitamin B12	$C_{63}H_{88}CoN_{14}O_{14}P$	3.7E-05	4.7E-08
P-aminobenzoic acid	$C_7H_7NO_2$	3.7E-04	4.7E-07
Calcium D(+) Pantothenate (Vit. B5)	$C_{18}H_{32}CaN_2O_{10}$	1.1E-04	1.3E-07
Thiamine Hydrochloride (Vit. B1)	$C_{12}H_{18}Cl_2N_4OS$	2.2E-04	2.8E-07
Pyridoxine-HCl (Vit. B6)	$C_8H_{12}ClNO_3$	4.9E-04	6.2E-07
Pyridoxamine-2HCl (Vit. B6)	$C_8H_{14}Cl_2N_2O_2$	1.0E-03	1.3E-06
Nicotinamide	$C_6H_6N_2O$	8.2E-04	1.1E-06
Nicotinic Acid (Niacin. Vit. B3)	$C_6H_5NO_2$	8.1E-04	1.0E-06
Riboflavin (Vit. B2)	$C_{17}H_{20}N_4O_6$	1.3E-04	1.7E-07
Folic Acid (Vit. B9)	$C_{19}H_{19}N_7O_6$	4.5E-05	5.8E-08
Lipoic Acid	$C_8H_{14}O_2S_2$	2.4E-04	3.1E-07
L-Ascorbic Acid (Vit. C)	$C_6H_8O_6$	5.7E-04	7.3E-07
Boric Acid	H_3BO_3	6.5E-04	8.3E-07
Manganese Sulphate Monohydrate	$MnSO_4$ x $1H_2O$	1.5E-04	2.0E-07
Cobalt (II) Chloride Hexahydrate	$CoCl_2$ x $6H_2O$	2.1E-04	2.7E-07
Nickel (II) Chloride Hexahydrate	$NiCl_2$ x $6H_2O$	1.1E-04	1.3E-07
Cupric Chloride Dihydrate	$CuCl_2$ x $2H_2O$	1.8E-04	2.3E-07
Zinc Chloride	$ZnCl_2$	2.9E-04	3.8E-07
Ammonium Molybdate	$(NH_4)_6Mo_7O_{24}$ x $4H_2O$	4.1E-05	5.2E-08
Aluminium Chloride Hydrate	$AlCl_3$ x H_2O	3.8E-04	4.8E-07
Sodium Selenite Pentahydrate	Na_2SeO_3 x $5H_2O$	5.7E-05	7.3E-08
Sodium Tungstate Dihydrate	Na_2WO_4 x $2H_2O$	6.1E-05	7.8E-08
Chloride	Cl^-	0	1.2E-01 ± 8.7E-03
Sulphate	SO_4^{2-}	0	7.5E-02 ± 1.5E-02
Ammonium	NH_4^+	0	< 1.3E-03 ± < 2.6E-04
Nitrite	NO_2^-	0	< 2.6E-04 ± < 1.8E-04
Nitrate	NO_3^-	0	1.7E-02 ± 1.7E-03
Aluminium	Al	0	< 4.0 E-04 ± < 8.0E-05
Calcium	Ca	0	1.7E-01 ± 1.7E-02
Copper	Cu	0	< 2.8E-04 ± < 5.7E-05
Iron	Fe	0	< 1.3E-04 ± < 2.6E-05
Potassium	K	0	1.7E-02 ± 1.7E-03
Magnesium	Mg	0	3.7E-02 ± 3.7E-03
Manganese	Mn	0	< 3.3E-05 ± < 3.3E-06
Sodium	Na	0	1.3E-01 ± 1.3E-02

[a] Sodium sulphate and hydrolysed casein served as precursors for sulphide and sulphur sources (Muyzer and Stams, 2008).

Table S2. Number of bacterial sequence reads remaining for each sample at each step of the DADA2 pipeline. *Input* – number of raw sequences; *Filtered* – number of sequences after filtering; *Denoised* – number of sequences after sample inference; *Merged* – number of sequences after merging the forward- and reverse reads; *Tabled* – reads after constructing the sequence table; *Nochim.* – number of reads remaining after removal of chimeras.

Sample	Sampling day	Input	Filtered	Denoised	Merged	Nochim.
**S001**	2	157488	9679	9679	9290	6855
**S002**	2	133544	6853	6853	6507	4937
**S003**	2	97806	7865	7865	7638	5802
**S004**	10	160003	10765	10765	10494	8029
**S005**	10	184813	11504	11504	11195	8604
**S006**	10	95056	7902	7902	7633	5934
**S007**	101	155128	11921	11921	11374	8560
**S008**	101	64595	5051	5051	4807	3736
**S009**	101	146621	11231	11231	10643	8020
**S010**	129	147860	10050	10050	9725	7740
**S011**	129	1018960	79711	79711	78405	59981
**S012**	129	102963	8816	8816	8608	6940
**S013**	178	226856	15396	15396	14993	12073
**S014**	178	123807	9647	9647	9545	8122
**S015**	178	290916	23000	23000	22600	17952
**S016**	214	188635	13183	13183	12970	10447
**S017**	214	130434	8379	8379	8253	6746
**S018**	214	114730	8519	8519	8382	6785
**S019**	228	162349	13403	13403	13213	10636
**S020**	228	203808	13230	13230	13031	10381
**S021**	228	95800	7227	7227	7105	5661
**S022**	260	168666	13036	13036	12809	10332
**S023**	260	106268	7287	7287	7137	5828
**S024**	260	143560	9136	9136	8994	7283
**S025**	304	157509	9486	9486	9334	7734
**S026**	304	186188	11171	11171	11013	9119
**S027**	304	125895	8737	8737	8568	7087
**S028**	332	87057	6670	6670	6551	5429
**S029**	332	134589	4605	4605	4499	3727
**S030**	332	281734	20401	20401	20186	16659
**S034**	381	120858	7447	7447	7234	6027
**S035**	381	141994	8800	8800	8593	7127
**S036**	381	157280	10875	10875	10687	8738
**S037**	472	168389	12675	12675	12386	10162
**S038**	472	175748	6139	6139	6055	4998
**S039**	472	195416	13881	13881	13623	11161

Table S3. Number of archaeal sequence reads remaining for each sample at each step of the DADA2 pipeline. *Input* – number of raw sequences; *Filtered* – number of sequences after filtering; *Denoised* – number of sequences after sample inference; *Merged* – number of sequences after merging the forward- and reverse reads; *Tabled* – reads after constructing the sequence table; *Nochim*. – number of reads remaining after removal of chimeras.

Sample	Sampling day	Input	Filtered	Denoised	Merged	Nochim.
**S001**	2	49002	18232	18232	1251	852
**S002**	2	39221	11579	11579	621	477
**S003**	2	69662	27097	27097	1669	1200
**S005**	10	45009	16152	16152	803	502
**S006**	10	93133	35043	35043	1950	1288
**S007**	101	82621	26438	26438	1339	1219
**S008**	101	30482	9797	9797	560	508
**S009**	101	35337	9665	9665	354	346
**S010**	129	27608	9151	9151	891	791
**S011**	129	44512	15131	15131	1895	1659
**S012**	129	71457	19600	19600	2113	1857
**S013**	178	38711	11849	11849	1373	1291
**S014**	178	51788	17246	17246	1864	1661
**S015**	178	40266	12881	12881	1783	1616
**S016**	214	62837	19329	19329	4551	3978
**S018**	214	36210	10378	10378	2676	2257
**S019**	228	59819	18291	18291	3057	2818
**S020**	228	67304	19354	19354	2848	2424
**S021**	228	55190	17916	17916	2741	2448
**S022**	260	57748	19226	19226	4357	3627
**S023**	260	48037	12650	12650	2796	2272
**S024**	260	62155	18105	18105	4315	3487
**S025**	304	57543	18742	18742	4389	4263
**S026**	304	53777	16828	16828	3123	2811
**S027**	304	85121	25704	25704	5223	4576
**S029**	332	53330	16670	16670	2172	1935
**S030**	332	54290	17313	17313	2413	2187
**S034**	381	41943	11459	11459	745	654
**S035**	381	41259	13417	13417	982	882
**S036**	381	69205	22908	22908	1602	1399
**S037**	472	67879	21849	21849	2732	2407
**S038**	472	82710	19524	19524	1804	1568
**S039**	472	46833	15378	15378	2247	1886

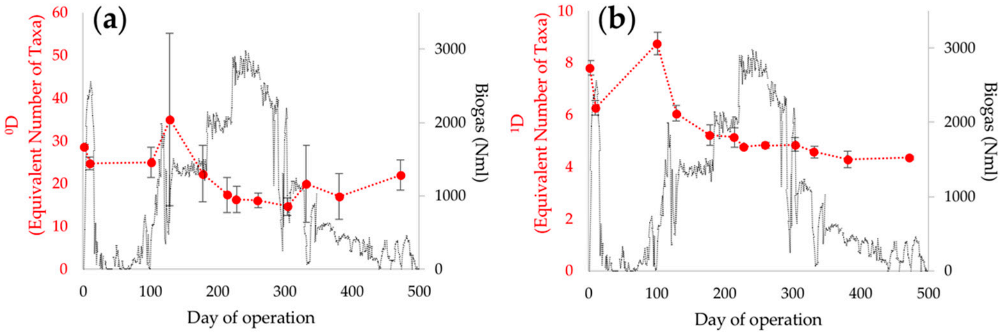

Figure S1. Hill diversity indices *⁰D* (a), and *¹D* (b) for bacterial 16S rRNA profiles together with biogas production of the reactor.

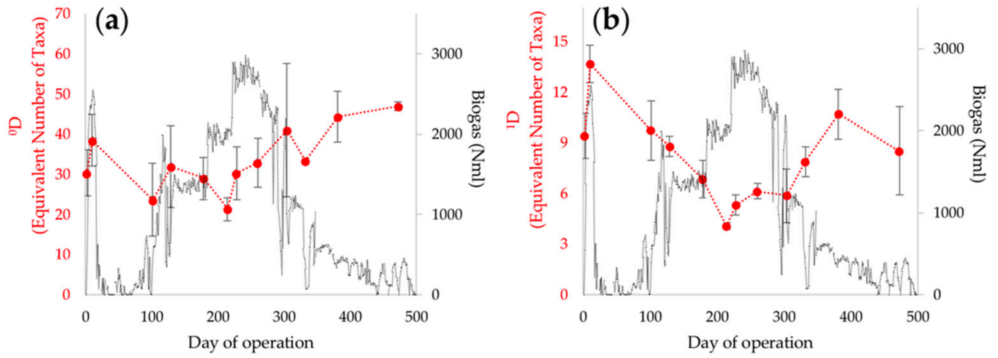

Figure S2. Hill diversity indices *⁰D* (a), and *¹D* (b) for archaeal 16S rRNA profiles together with biogas production of the reactor.

Supplementary references:

1. Callahan, B.J., McMurdie, P.J., Rosen, M.J., Han, A.W., Johnson, A.J.A., Holmes, S.P., 2016. DADA2: High resolution sample inference from Illumina amplicon data. Nat. Methods 13, 581–583. https://doi.org/10.1038/nmeth.3869
2. Martin, M., 2011. Cutadapt removes adapter sequences from high-throughput sequencing reads. EMBnet.journal 17, 10. https://doi.org/10.14806/ej.17.1.200
3. McMurdie, P.J., Holmes, S., 2013. Phyloseq: An R Package for Reproducible Interactive Analysis and Graphics of Microbiome Census Data. PLoS One 8. https://doi.org/10.1371/journal.pone.0061217
4. Muyzer, G., Stams, A.J.M., 2008. The ecology and biotechnology of sulphate-reducing bacteria. Nat. Rev. Microbiol. 6, 441–454. https://doi.org/10.1038/nrmicro1892
